# A Rare Diagnosis: Anti-HMGCR Antibody-mediated Necrotizing Myositis

**DOI:** 10.51894/001c.144658

**Published:** 2025-09-30

**Authors:** Shamaiza Waqas

**Affiliations:** 1 Department of Internal Medicine Henry Ford Warren

## 131

### INTRODUCTION

Immune-mediated necrotizing myositis (NM) is a rare cause of proximal and symmetric muscle weakness. Immune-mediated NM represents approximately 20% of all diagnosed autoimmune myopathies. The pathogenesis of this condition is largely unknown but it is thought to be triggered by viral infections, cancers, connective tissue diseases, and importantly: medications, such as statins. Anti-3-hydroxy-3-methylglutaryl-coenzyme A reductase (anti-HMGCR) autoantibodies can be found in autoantibody-positive cases of immune-mediated NM.

### CASE DESCRIPTION

The patient was a 60-year-old female with a past medical history of diabetes mellitus, hypertension, hypercholesterolemia, and hypothyroidism. Medications included statin,lisinopril, Levemir, and levothyroxine. She presented with upper and lower extremity weakness and aches. On physical examination, the patient showed proximal muscle weakness. Vital signs were stable. Her initial blood work showed an elevated CPK level at 4,852 IU/L and an elevated HMG-CoA reductase inhibitor antibody titer at >200 CU. Electromyography showed axonal sensorimotor peripheral neuropathy and evidence of myopathy without clear evidence of segmental necrosis. Muscle biopsy showed necrotizing myopathy with regeneration and degeneration of muscle fibers and few macrophages. The patient was started on Methotrexate 2.5 mg 7 tabs weekly and Prednisone 60 mg/day, CPK improved to 1019 but her weakness worsened. IVIG was started and Prednisone tapered off. CPK normalized at 211 IU/L and the patient displayed improved muscle strength. Lumbar puncture done to assess for CIDP, showed elevated WBCs, mildly elevated protein at 53, and a minimally increased IgG CSF at 3.5 (normal: 3.4).

### DISCUSSION

Anti-HMGCR-mediated NM following statin exposure requires prompt evaluation and treatment, highlighting the importance of differentiating anti-HMGCR-mediated NM from the more common statin-induced toxic myopathy. Measuring serial CPK levels in suspected NM patients allows practitioners to monitor disease progression but it is important to note that CPK levels may be normal in undiagnosed, chronic immune-mediated NM patients, as necrotic muscle fibers are replaced with connective tissue. Management of anti-HMGCR-mediated NM requires a multifaceted approach, including immunotherapy, physical therapy, pulmonary function tests, and malignancy screening, to avoid long-term disability.

